# Sensorless based SVPWM-DTC of AFPMSM for electric vehicles

**DOI:** 10.1038/s41598-022-12825-x

**Published:** 2022-05-30

**Authors:** Saber M. Saleh, Amir Y. Hassan

**Affiliations:** 1 Department of Electrical Engineering, Faculty of Engineering, Fayoum University, Fayoum, Egypt; 2 Department of Power Electronics and Energy Conversion, Electronics Research Institute, Cairo, Egypt

**Keywords:** Engineering, Electrical and electronic engineering

## Abstract

AFPMSM is lighter, has a higher power-to-weight ratio, is shorter in length, is less expensive, and has a higher efficiency than the radial flux motor. Then AFPMSM is more suitable for driving the EV than radial flux motor. The proposed technique in this paper is the sensorless-based SVPWM-DTC of AFPMSM to drive electric vehicles. Sensorless research becomes more important in this circumstance since the axial motor can be placed inside the vehicle tire due to its condensed size and shape similar to the tires. DTC provides less fluctuation for the driver during driving for safety and comfort. SVPWM is preferred for its high performance. When measuring speed using a sensorless estimator, sensor inaccuracy is minimized, and the AFPMS motor can be mounted inside the tire. The control system is tested using two EVs driving cycles, and the results promise high performance. NEDC and HWFET driving cycles are used to test the proposed control scheme in 100 times less than the actual driving cycles’ time to test the coherence of the sensorless estimator. The results demonstrate that the proposed technique is valid for real-time applications with high-performance, minimum torque fluctuations, and minimum transient and steady-state errors.

## Introduction

EVs are better for the environment, but when power is a renewable resource, fuel vehicles are not. Also, it requires less frequent and less costly maintenance. EVs are quieter than fuel-powered vehicles. Owners of electric cars are eligible for tax credits, and there are even particular highway paths for EVs in some places. Fuel-powered autos have a longer driving distance. Charging time for EVs can be longer, but less expensive than fuel-powered vehicles. The availability of charging stations can be limited, and there are fewer models available^1^. DC Series Motor, Brushless DC Motor, Permanent Magnet Synchronous Motor (PMSM), Three Phase AC Induction Motors, and Switched Reluctance Motors are the motors used to propel EVs^2,3^. PMSM are recommended for driving EVs because they may give a high efficiency, and ruggedness, as well as a high torque-to-current ratio^4^. The AFPMSM is better than the radial flux one for driving vehicles for higher efficiency is, lower weight, shorter length, lower cost, and better torque^5,6^.

The AFPMSM idea encompasses a wide variety of architectures based on rotor and stator combinations. Single-rotor single-stator, double-rotor single-stator, single-rotor double-stator, and multi-rotor multi-stator are the four different types of AFPMSM^7,8^. They can have a slotted or slotless stator structure and permanent magnets on the rotor-disk surface or within the rotor disc. The main flux passes axially through the rotor disc or circumferentially around it. The rotor of the surface-mounted construction is extremely thin, particularly if the magnets are positioned inside a non-ferromagnetic rotor core. When permanent magnets are buried in the rotor disc, a substantially thicker rotor disc is required, lowering machine power density while the machine stator structure stays the same. Because its rotor lacks iron, axial flux interior rotor (AFIR) architecture provides a very high power-to-inertia ratio, making it ideal for applications requiring low inertia^8^.

Low cost and high reliability make controlled electric motors attractive. Sensorless means no electrical sensors to read the motor speed. PMSMs are being employed more than ever before in servo control systems. Speed sensors provide feedback control for servo control systems, resulting in excellent performance. For applications such as an EV, shaft sensors have disadvantages, such as increased system costs, motor size, and reliability. Therefore, extensive research has been conducted on eliminating speed sensors in servomotor systems to overcome these difficulties^9–14^. This study employs a sensorless estimator guided by a reference speed to overcome changes in the driver reference speed. DTC is one of the ways used in variable-frequency drives to control the speed of three-phase AC electric motors. As a result of measuring the motor's voltage and current, a rough approximation of the motor's magnetic flux and torque can be calculated^15^. SVPWM technique is used after the DTC to identify the pulse-width modulated signals for the inverter switches to provide the required three-phase voltages for the motors^16^.

**Figure 1:**
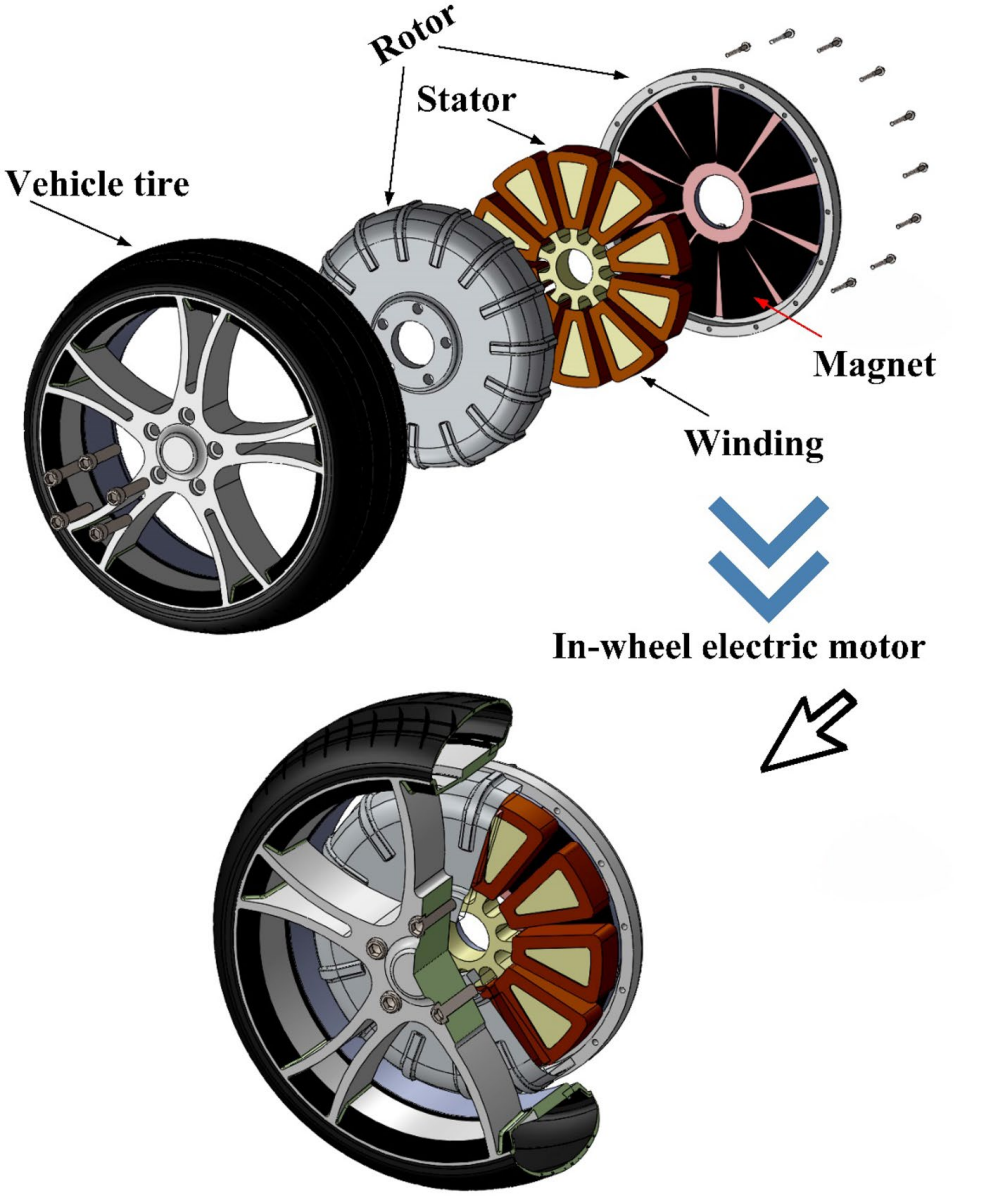
In-wheel constructed AFPMS motor for electric vehicle application.

The proposed technique uses sensorless based DTC-SVPWM control strategy for AFPMSM motor driving system for EV. Torque variation when driving can have an impact on the drivers’ comfort as well as their safety. For safety and comfort, DTC ensures less fluctuation for the driver while driving. SVPWM is recommended because its higher PF, lower THD rate, and lower switching losses, high efficiency, and good performance are achieved. A sensorless estimator is used to decreased sensor inaccuracy while observing speed, and the motor can also be inserted inside the tire using the sensorless estimator. Single-stator single-rotor, single-stator double-rotor, and double-stator single-rotor structures are the structures that come closest to being acceptable for usage in-wheel. But, the single-rotor single-stator structure, particularly the surface-mounted structure, is the more suitable one due to its small thickness. The AFPMSM can be mounted in-wheel, as shown in Fig. 1. Two driving cycles were used to test the total control system, with promising results. The paper items are AFPMSM model, DTC, SVPWM, straight-line guided by the reference speed sensorless estimator, simulation and driving cycles of EV results, and conclusion.

## Axial flux permanent magnet synchronous motor model

The AFPMSM is an advanced technology that can be used for multi-megawatt applications and is suitable for applications that required high torque with low speed like electric tractions. Axial flux motors use less material and can also deliver higher power density than that of radial flux motors, and this means the axial flux motors suitable for an EV^5^. The axial flux motors are better than the radial flux motors because the radial flux motors are heavier than axial flux motors, longer than axial motor length, and the axial flux motor efficacy is higher^6^.

The AFPMSM’s dq0 reference frame equations under assumptions of neglected saturation effect, losses generated by hysteresis, eddy currents, and stray. The studied AFPMSM not containing a salient pole effect, so ${L}_{d}$ equal to ${L}_{q},$ and Sinusoidal back-EMF is as follow^17–20^:

1 $${\mathcal{U}}_{\mathrm{da}}={\mathrm{R}}_{\mathrm{sa}} {\fancyscript{i}}_{\mathrm{da}}+\frac{\mathrm{d}{\uppsi }_{\mathrm{da}}}{\mathrm{dt}}-{P\omega }_{r}{\psi }_{qa}$$

2 $${\mathcal{U}}_{\mathrm{qa}}={\mathrm{R}}_{\mathrm{sa}} {\fancyscript{i}}_{\mathrm{qa}}+\frac{\mathrm{d}{\uppsi }_{\mathrm{qa}}}{\mathrm{dt}}+{P\omega }_{r}{\psi }_{da}$$

where ${\mathcal{U}}_{\mathrm{da}} ,{\mathcal{U}}_{\mathrm{qa}}$ is the $\mathrm{stator voltage in}$ d–q axis, ${\fancyscript{i}}_{\mathrm{da}} , {\fancyscript{i}}_{\mathrm{qa}}$ is the $\mathrm{stator current in}$ d–q axis, ${\psi }_{qa} , {\psi }_{da} \mathrm{is the} \mathrm{flux in}$ d–q axis, ${\mathrm{R}}_{\mathrm{sa}}$ is the $\mathrm{resistance of stator windings}.$

Where direct-quadrature stator flux equations:

3 $${\psi }_{da}={L}_{da} {\fancyscript{i}}_{da}+{\psi }_{f}$$

4 $${\psi }_{qa}={L}_{qa} {\fancyscript{i}}_{qa}$$

where ${L}_{da}$ is the D $\mathrm{axis \, winding \, inductance}$, ${L}_{qa}\mathrm{ is \, the}$ Q $\mathrm{axis \,winding \, inductance}.$

* Torque equation:

5 $${T}_{e}={\frac{n}{2} P i}_{qa}\left[{\psi }_{f}+\left({L}_{da}-{L}_{qa}\right){i}_{da}\right]$$

where n is the $\mathrm{number \, of \, phases}, {\uppsi }_{\mathrm{f}}\mathrm{ is \, the \, flux \, of \, the \, field \, of \, rotor}$ windings.

6 $${\mathrm{T}}_{\mathrm{e}}={\frac{\mathrm{n}}{2}\mathrm{ P i}}_{\mathrm{qa}}{\uppsi }_{\mathrm{f}}$$

The torque can be controlled by changing ${\mathrm{i}}_{\mathrm{qa}}$ for ${L}_{da}={L}_{qa}$

## Direct torque control (DTC)

The speed of three-phase AC electric motors can be adjusted using DTC in variable-frequency drives. Voltage and current measurements on the electric motor can yield an approximation of the magnetic flux and torque^21,22^.

7 $${T}_{e}={P i}_{sa}{\psi }_{sa}$$

where $${T}_{e}\mathrm{ is \, the \,electromagnetic \, torque}$$, P $$\mathrm{is \,the \, pole}-\mathrm{pairs \,number}$$, $${\psi }_{sa}$$ $$\mathrm{is \,the \,flux \,of \,the \,field \, of \, stator \, windings}$$, $${i}_{sa}$$ $$\mathrm{is \,the \,stator \, current \, vector}.$$

8 $${T}_{e}-{T}_{l}-\mathrm{\rm B}{\upomega }_{\mathrm{r}}=\mathrm{J}\frac{d{\omega }_{r}}{dt}$$

where $${T}_{l}\mathrm{ is \, the \, torque \, of \, load}$$, $${\upomega }_{\mathrm{r}}\mathrm{ is \, the \, rotor \, angular \, velocity}$$, $$\mathrm{\rm B}$$ $$\mathrm{is \, the \, damping \, coefficient}$$, $$J \mathrm{is \, the \, inertia \, moment \, for }AFPMSM.$$

The electromagnetic torque could be written in the form of

9 $${T}_{e}={\frac{n}{2} \frac{P}{{L}_{da}} i}_{qa }{\psi }_{sa} {\psi }_{f}\mathrm{sin}\delta + \frac{n}{4}\frac{{L}_{da}-{L}_{qa}}{{L}_{qa}{L}_{da}} {{\psi }_{sa}}^{2}\mathrm{sin}2\delta$$

where $\delta$ is the torque angle.

For ${L}_{d}$ equal to ${L}_{q}$ and the torque equation will be:

10 $${T}_{e}={\frac{n}{2} \frac{P}{{L}_{da}} i}_{qa }{\psi }_{sa} {\psi }_{f}\mathrm{sin}\delta$$

From this equation, it is clear that changing electromagnetic torque depends upon changing angle δ.

Flux estimation equations are Eqs. (3) and (4) and the AFPMSM stator flux given by:

11 $${\psi }_{sa}=\sqrt{{({\psi }_{qa})}^{2}+{({\psi }_{da})}^{2}}$$

The AFPMSM reference flux is calculated from the following equation^21,22^.

12 $$\left|\uppsi \begin{array}{c}*\\ s\end{array}\right|=\sqrt{\uppsi \begin{array}{c}2\\ f\end{array}+{\left(\frac{2}{3}\frac{T\begin{array}{c}*\\ e\end{array}{L}_{s}}{p{\psi }_{f}}\right)}^{2}}$$

A trial-and-error approach is used to evaluate the gains of the PI controller and the constants listed in Table 1^23^.

**Table 1:** Constant values of the PI controllers.

Case	${K}_{P}$	${K}_{i}$
Speed	20	45
Flux	1	75
Torque	150	100

13 $$\mathrm{T}\begin{array}{c}*\\ e\end{array}={k}_{p}e+{k}_{i}\int edt$$

14 $$e={\omega }_{ref}-\upomega$$

15 $${U}_{d}={k}_{p}e+{k}_{i}\int edt$$

16 $$e=\uppsi \begin{array}{c}*\\ s\end{array}-{\uppsi }_{s}$$

17 $${U}_{q}={k}_{p}e+{k}_{i}\int edt$$

18 $$e=\mathrm{T}\begin{array}{c}*\\ e\end{array}-{\mathrm{T}}_{e}$$

## Space vector pulse width modulation (SVPWM) inverter

PWM waveform generation, switching time calculation model, and sector selection are all part of the SVPWM model in MATLAB^24–26^. The switching frequency of pulse width is 20,000 Hz, the DC voltage is 250-V, and the reference speed is 300 rpm.

* Reference voltage and angle

19 $$\left|\begin{array}{c}{u}_{d}\\ {u}_{q}\end{array}\right|=\frac{2}{3}\left|\begin{array}{ccc}1& -0.5& -0.5\\ 0& \frac{\sqrt{3}}{2}& -\frac{\sqrt{3}}{2}\end{array}\right|\left|\begin{array}{c}{u}_{an}\\ {u}_{bn}\\ {u}_{cn}\end{array}\right|$$

20 $${u}_{ref}=\sqrt{u\begin{array}{c}2\\ d\end{array}+u\begin{array}{c}2\\ q\end{array}}$$

21 $$\alpha ={tan}^{-1}\left(\frac{{u}_{q}}{{u}_{d}}\right)$$

* Conversion time in any sector

${T}_{s}=\frac{1}{f}$, $f$ is the fixed clock frequency

22 $${T}_{1}=\frac{\sqrt{3}{T}_{s}{V}_{ref}}{{V}_{dc}}\mathrm{sin}\left(\frac{n}{3}\pi -\alpha \right)$$

23 $${T}_{2}=\frac{\sqrt{3}{T}_{s}{V}_{ref}}{{V}_{dc}}\mathrm{sin}\left(\alpha -\frac{n-1}{3}\pi \right)$$

24 $${T}_{0}={T}_{f}-{T}_{1}-{T}_{2}$$

where $n$ is the sector from 1 to 6, $0\le \alpha \le {60}^{o}$

## Straight-line guided by the reference speed sensorless estimator

**Figure 2:**
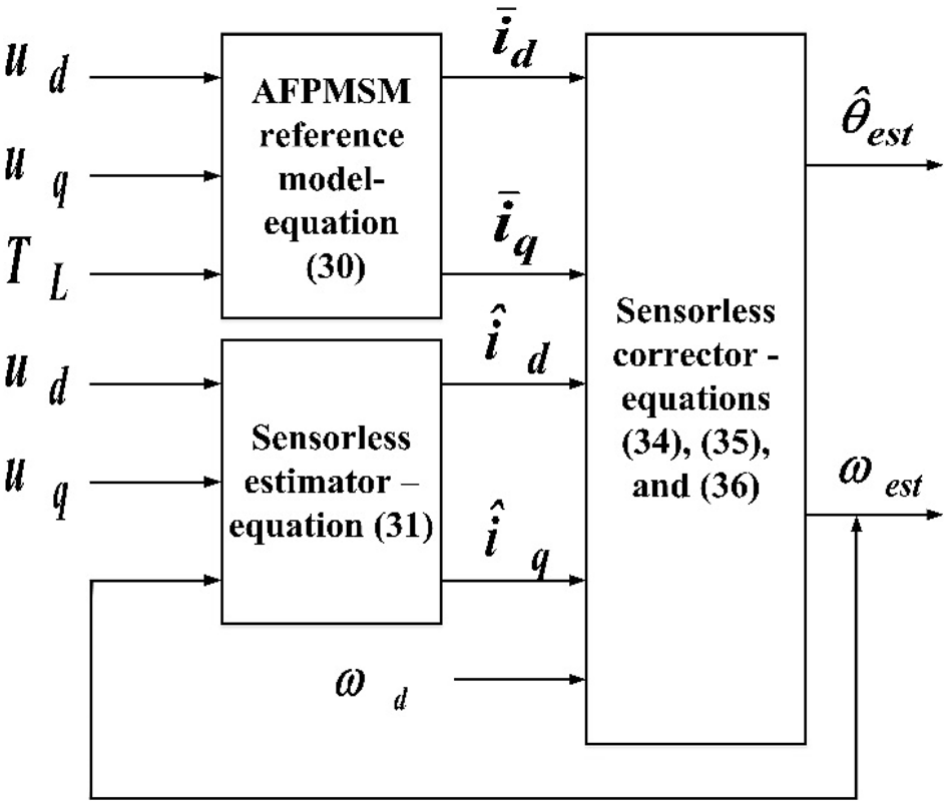
The proposed straight-line guided by the reference speed sensorless estimator.

The proposed straight-line guided by the reference speed sensorless estimator is consists of the AFPMSM reference model, sensorless estimator, and sensorless corrector, as shown in Fig. 2.

✓ Axial flux permanent magnet synchronous motor reference model is explained using Eqs. (1) to (6).

From Eqs. (1), (3), and (4) the stator voltage equation in d-axis become

25 $${\mathcal{U}}_{\mathrm{d}}=\mathrm{R }{\fancyscript{i}}_{\mathrm{d}}+\frac{\mathrm{d}(L {\fancyscript{i}}_{d}+{\psi }_{f})}{\mathrm{dt}}-{P\omega }_{r}(L {\fancyscript{i}}_{q})$$

Taking ${\omega }_{e}={P\omega }_{r}$ then the current can be driven as:

26 $$\frac{{d\widehat{i}}_{d}}{dt}=-\frac{R}{L}{\widehat{i}}_{d}+{\omega }_{e}{\widehat{i}}_{q}+\frac{{u}_{d}}{L}$$

From Eqs. (2), (3), and (4) the stator voltage equation in q-axis become

27 $${\mathcal{U}}_{\mathrm{q}}={\mathrm{R}}_{\mathrm{s}} {\fancyscript{i}}_{\mathrm{q}}+\frac{\mathrm{d}(L {\fancyscript{i}}_{q})}{\mathrm{dt}}+{P\omega }_{r}(L {\fancyscript{i}}_{d}+{\psi }_{f})$$

Again, by taking ${\omega }_{e}={P\omega }_{r}$ then the current can be driven as:

28 $$\frac{{d\widehat{i}}_{q}}{dt}=-\frac{R}{L}{\widehat{i}}_{q}-{\omega }_{e}{\widehat{i}}_{d}-\frac{{\uppsi }_{\mathrm{f}}}{L}{\omega }_{e}+\frac{{u}_{q}}{L}$$

The AFPMSM reference model stator current from Eqs. (26) and (28) is rewritten as the state variable as:

29 $$\frac{d}{dt}\left[\begin{array}{c}{i}_{d}+\frac{{\uppsi }_{\mathrm{f}}}{L}\\ {i}_{q}\end{array}\right]=\left[\begin{array}{cc}\frac{-R}{L}& {\omega }_{e}\\ {-\omega }_{e}& \frac{-R}{L}\end{array}\right]\left[\begin{array}{c}{i}_{d}+\frac{{\uppsi }_{\mathrm{f}}}{L}\\ {i}_{q}\end{array}\right]+\left[\begin{array}{c}\frac{{u}_{d}}{L}+\frac{R{\uppsi }_{\mathrm{f}}}{{L}^{2}}\\ \frac{{u}_{q}}{L}\end{array}\right]$$

For $${\overline{i} }_{d} ={i}_{d}+\frac{{\uppsi }_{\mathrm{f}}}{L}$$ , $${\overline{i} }_{q} ={i}_{q}$$, $${\overline{u} }_{d} ={u}_{d}+\frac{{\mathrm{R\psi }}_{\mathrm{f}}}{L}$$ , and $${\overline{u} }_{q} ={u}_{q}$$,

30 $$\therefore \frac{d}{dt}\left[\begin{array}{c}{\overline{i} }_{d} \\ {\overline{i} }_{q} \end{array}\right]=\left[\begin{array}{cc}\frac{-R}{L}& {\omega }_{e}\\ {-\omega }_{e}& \frac{-R}{L}\end{array}\right]\left[\begin{array}{c}{\overline{i} }_{d} \\ {\overline{i} }_{q} \end{array}\right]+\frac{1}{L}\left[\begin{array}{c}{\overline{u} }_{d} \\ {\overline{u} }_{q}\end{array}\right]$$

where ${\overline{i} }_{d}$ and ${\overline{i} }_{q}$ are the reference direct and quadrature current respectively, ${\omega }_{e}$ is the angular speed.

✓ Sensorless estimator

Replacing the reference value with estimated value and obtaining (Eq. 31):

31 $$\therefore \frac{d}{dt}\left[\begin{array}{c}{\widehat{i}}_{d} \\ {\widehat{i}}_{q} \end{array}\right]=\left[\begin{array}{cc}\frac{-R}{L}& {\widehat{\omega }}_{e}\\ {-{\widehat{\omega }}}_{e}& \frac{-R}{L}\end{array}\right]\left[\begin{array}{c}{\widehat{i}}_{d} \\ {\widehat{i}}_{q} \end{array}\right]+\frac{1}{L}\left[\begin{array}{c}{\widehat{u}}_{d} \\ {\widehat{u}}_{q}\end{array}\right]$$

where ${\widehat{i}}_{d}$ and ${\widehat{i}}_{q}$ are the estimated direct and quadrature current respectively, ${\omega }_{e}$ is the angular speed.

✓ Sensorless corrector

The error the reference and estimated currents is:

32 $${e}_{d}={\overline{i} }_{d}-{\widehat{i}}_{d }, {e}_{q}={\overline{i} }_{q}-{\widehat{i}}_{q}$$

Subtracting Eqs. (30) and (31), the error between the reference and the estimated currents is:

33 $$\frac{d}{dt}\left[\begin{array}{c}{e}_{d} \\ {e}_{q} \end{array}\right]=\left[\begin{array}{cc}\frac{-R}{L}& {\omega }_{e}\\ {-\omega }_{e}& \frac{-R}{L}\end{array}\right]\left[\begin{array}{c}{e}_{d} \\ {e}_{q} \end{array}\right]-({\widehat{\omega }}_{e}-{\omega }_{e})\left[\begin{array}{cc}0& 1\\ -1& 0\end{array}\right]\left[\begin{array}{c}{\widehat{i}}_{d} \\ {\widehat{i}}_{q}\end{array}\right]$$

**Figure 3:**
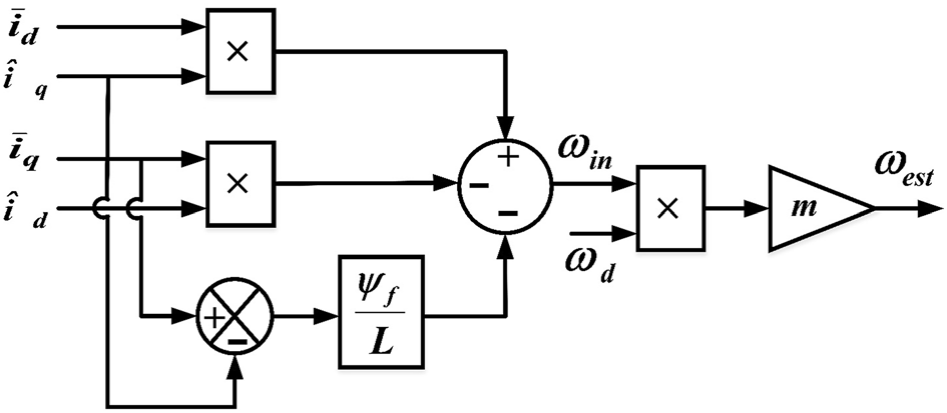
Schematic diagram of the proposed sensorless corrector.

The forward channel transfer function matrix is easily demonstrated to be a real matrix that is just positive. The electrical angular velocity adaptive law can then be determined using Popov hyper-stability theory by solving the Popov integral inequality^10^. The proposed sensorless speed can be estimated as Eq. (34) and the schematic diagram in Fig. 3.

34 $${\omega }_{in}=\left\{\left({\widehat{i}}_{q}\times {\overline{i} }_{d}\right)-\left({\widehat{i}}_{d}\times {\overline{i} }_{q}\right)\right\}-\frac{{\uppsi }_{\mathrm{f}}}{L}({\overline{i} }_{q} -{\widehat{i}}_{q})$$

where ${\omega }_{in}$ is the initial estimated speed without adjustment.

The relation between the estimated speed and the multiplying of demand speed and the initial estimated speed is assumed to be seen as a straight-line equation as follow:

35 $${\omega }_{est}=m\times {\omega }_{in}\times {\omega }_{d}$$

where ${\omega }_{est}and {\omega }_{d}$ are the estimated speed and demand speed, respectively. Also, $m$ is the slope of the straight-line.

At the ${\omega }_{d}=300 \mathrm{rpm}$, the initial estimated speed without adjustment is found to be ${\omega }_{in}=10000 \mathrm{rpm}$ then, the slope is taken to be 10,000 to produce ${\omega }_{est}=300 \mathrm{rpm}$.

The straight-line guided by the reference speed sensorless estimator is used to overcome changes in the drive's reference speed value by using the reference speed setting as a decision parameter.

Integrating the estimated speed as follows yields the rotor position:

36 $$\widehat{\theta }={\int }_{0}^{t}{\omega }_{est} dt$$

## Simulation and driving cycles of electric vehicle results

**Figure 4:**
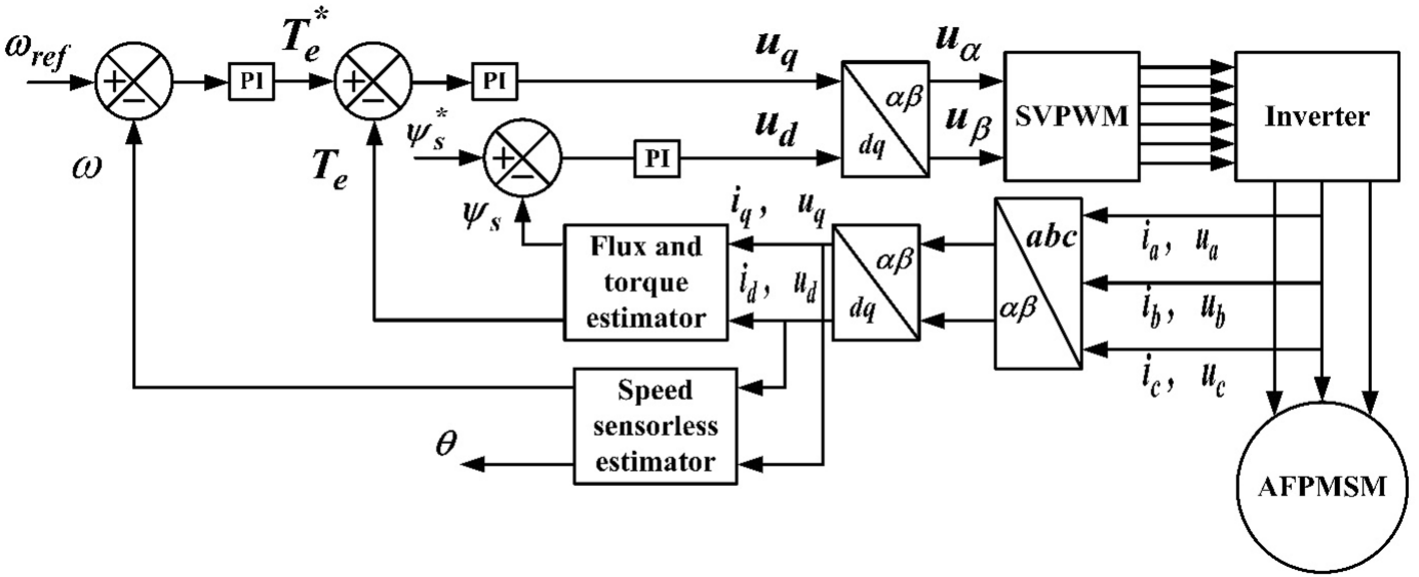
Sensorless DTC block diagram with SVPWM for AFPMSM.

The control block diagram of the proposed control sensorless-based scheme of axial flux permanent magnet motor is shown in Fig. 4. The Axial flux motor parameters are listed in Table 2.

**Table 2:** Axial flux motor parameters.

Parameter	Value
Pole pairs number	2
Stator resistance ${\mathrm{R}}_{\mathrm{sa}}$ (Ohm)	0.2
Stator inductance ${\mathrm{L}}_{\mathrm{Sa}}$ (mH)	8.5
Rotor magnetic flux ${\uppsi }_{\mathrm{f}}$ (Wb)	0.175
VDC dc-voltage (volt)	250
Damping coefficient $\mathrm{\rm B}$	0.005
Inertia moment $\mathrm{J}$	0.089
Rated speed (rpm)	300
Rated torque N m	11

**Figure 5:**
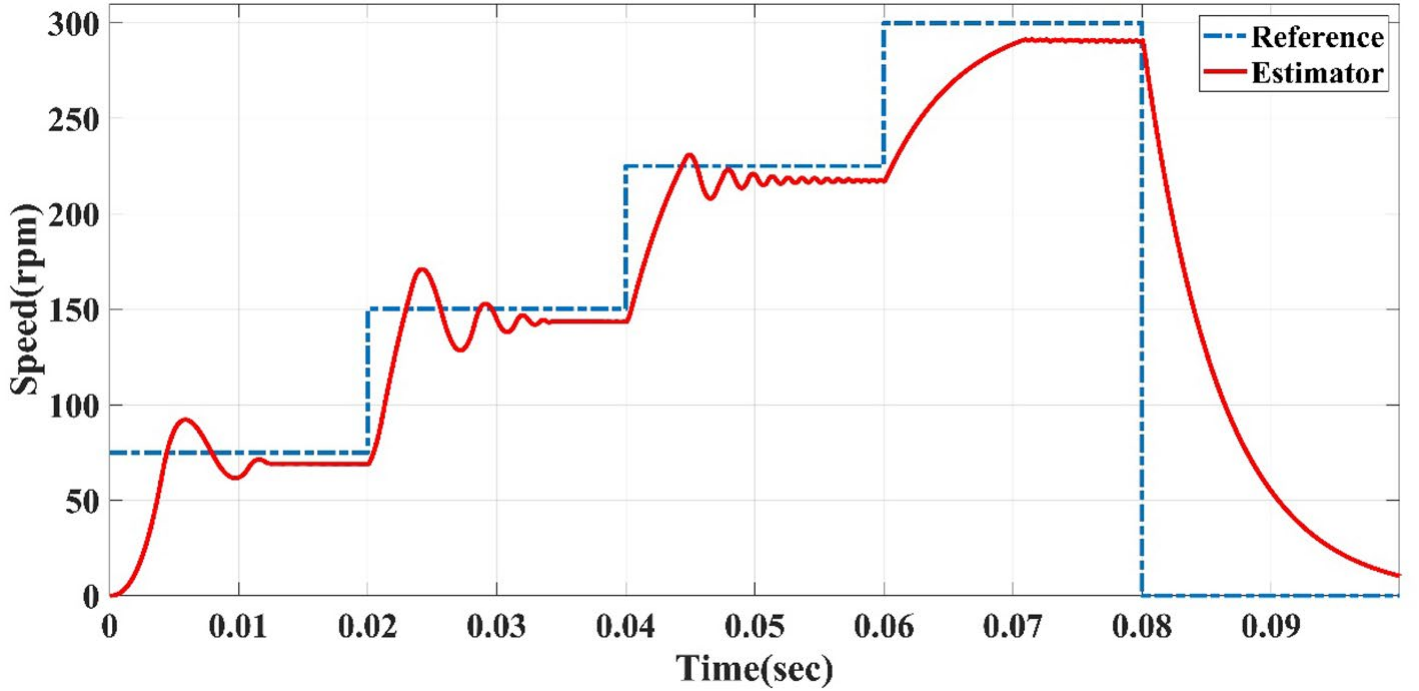
Reference speed cycle at 0.02-s interval and straight-line guided by reference speed sensorless estimator of AFPMSM.

**Figure 6:**
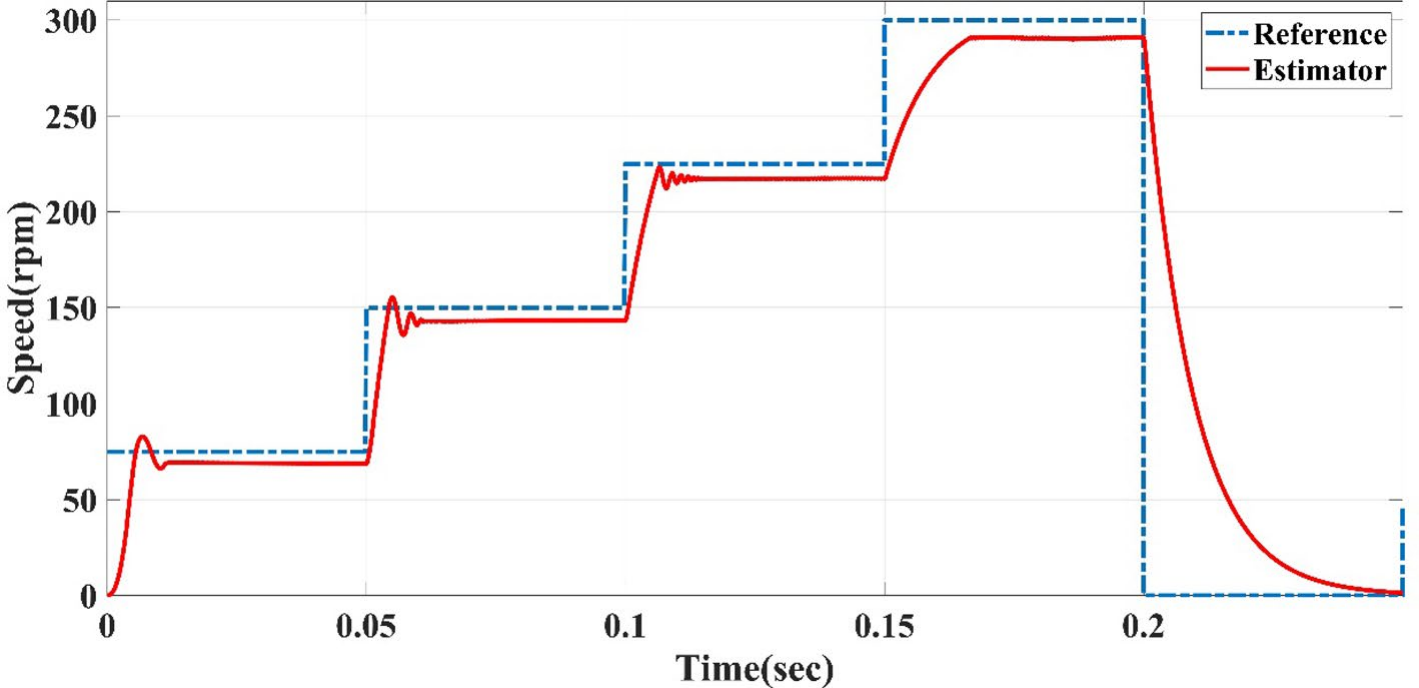
Reference speed cycle at 0.05-s interval and straight-line guided by reference speed sensorless estimator of AFPMSM.

**Figure 7:**
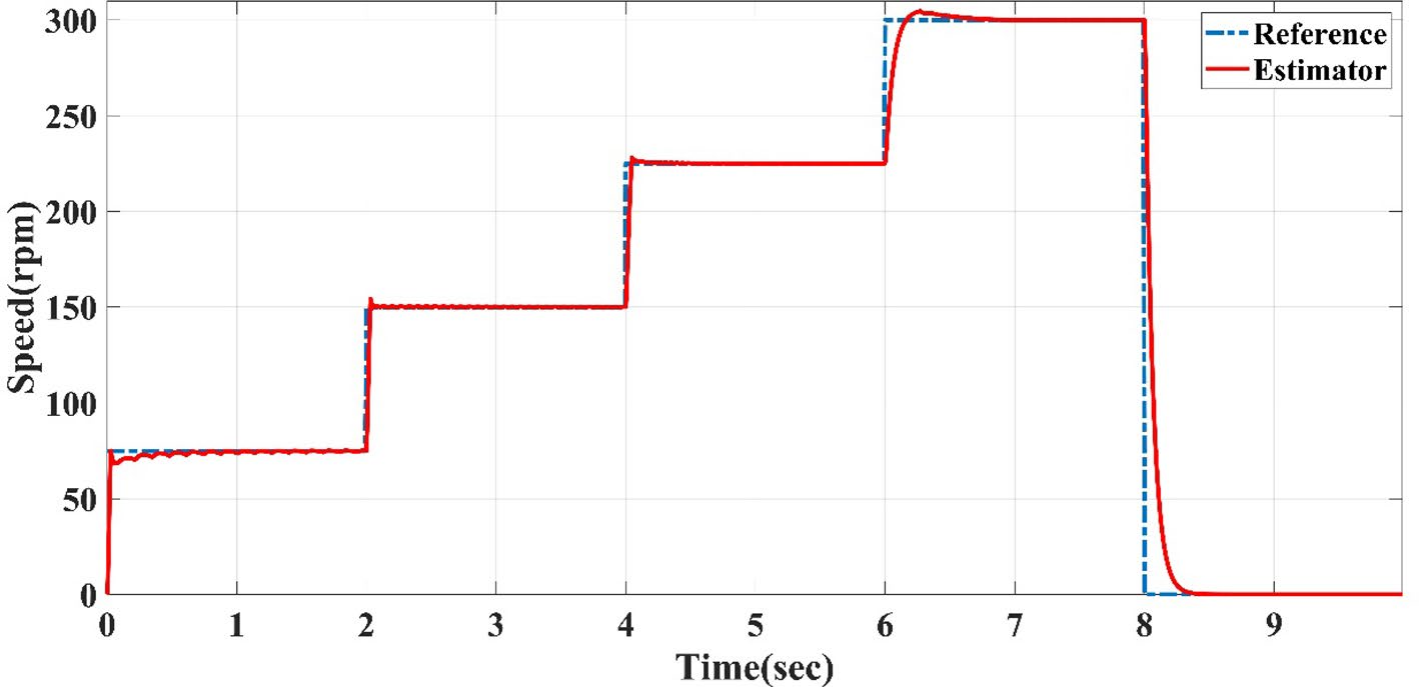
Reference speed cycle at 2-s interval and straight-line guided by reference speed sensorless estimator of AFPMSM.

The straight-line guided by the reference speed sensorless estimator in case of variable reference speed at full load, at different time intervals of 0.02 s, 0.05 s, and 2 s, the reference speed cycle is set to 75, 150, 0, 225, 300, 75 and 0 rpm. The straight-line guided by the reference speed sensorless estimator is tested to follow the reference speed cycle. The results of Figs. 5, 6 and 7 show the sensorless estimator reaches the set values of reference speeds with minimum overshoot (0.11%), slight steady-state errors (0.22%), and little rise time (0.05 s) as a steady-state and transient. The previous speed Figures show that this control system achieves a quick response and fluctuation as little as possible.

**Figure 8:**
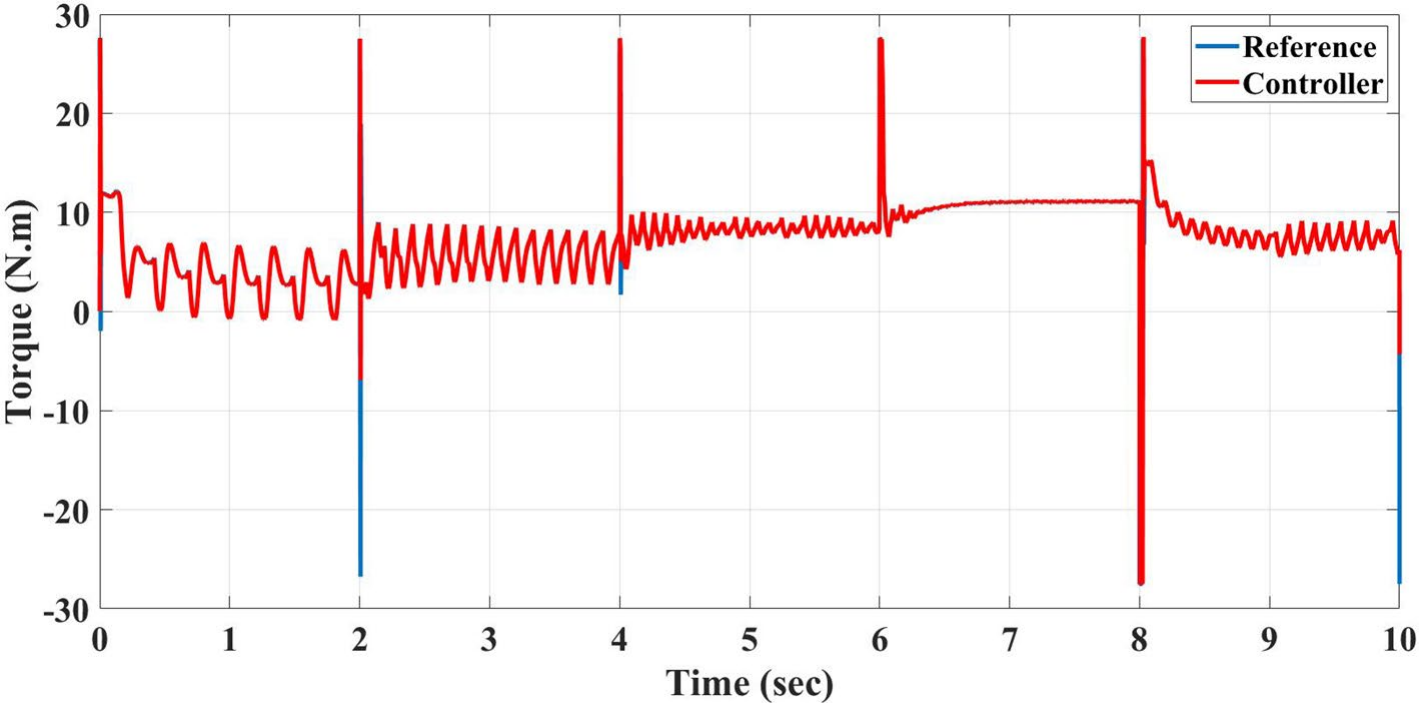
The AFPMSM torque response to the reference torque of DTC control.

**Figure 9:**
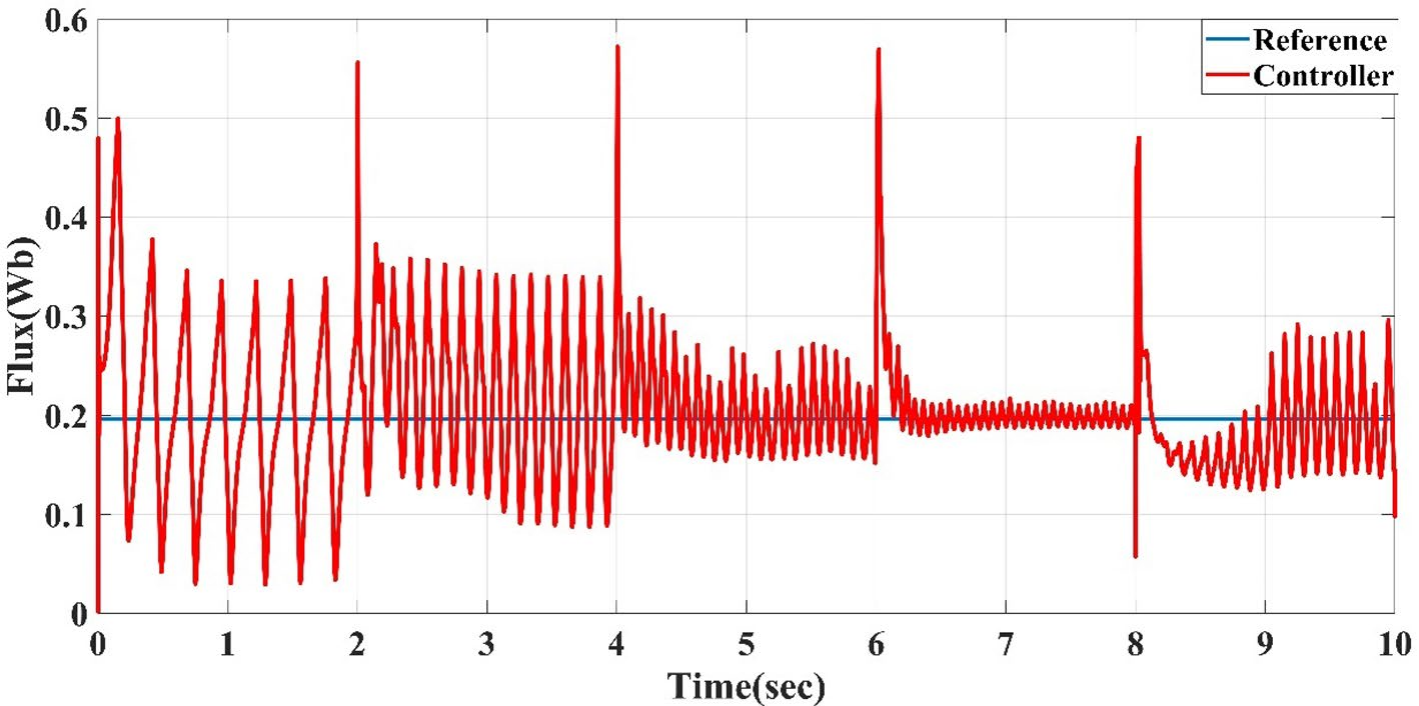
The AFPMSM flux response to the reference flux of DTC control.

**Figure 10:**
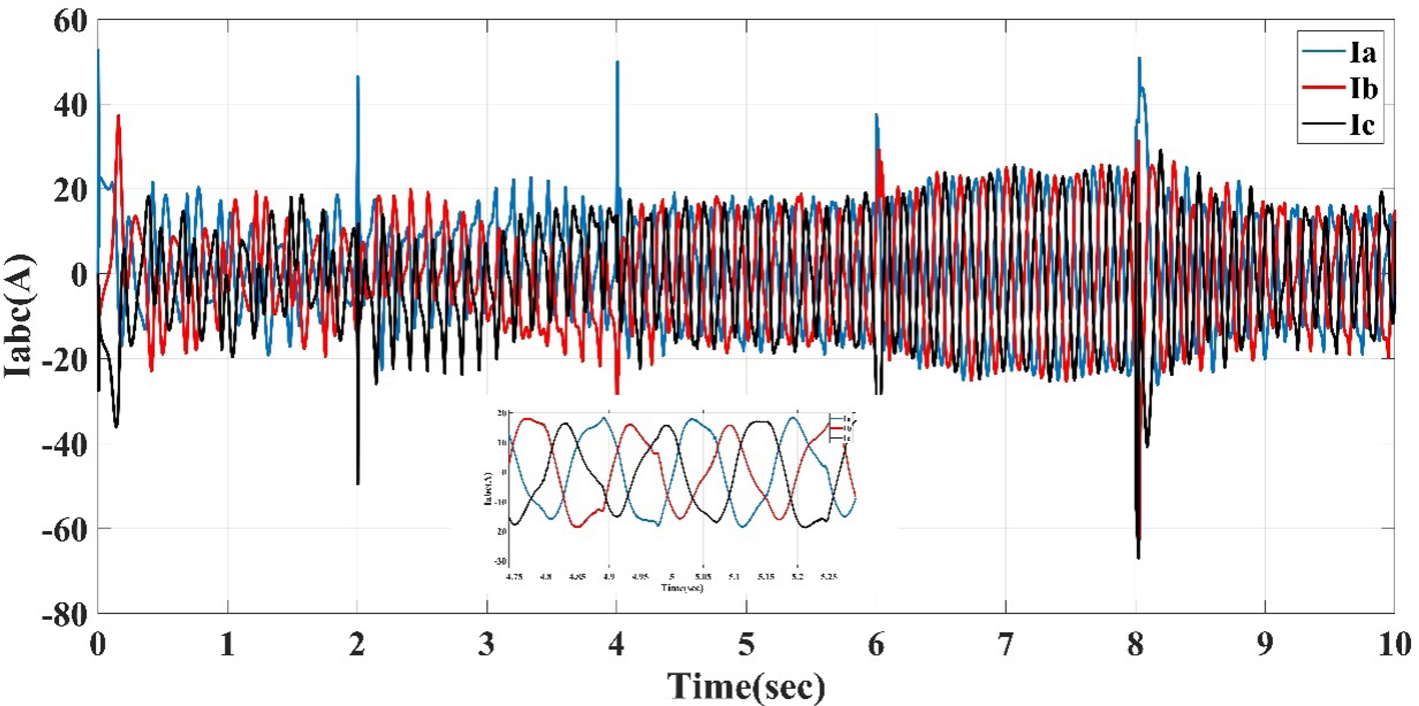
The AFPMSM instantaneous current at full load torque of 11 N m.

**Figure 11:**
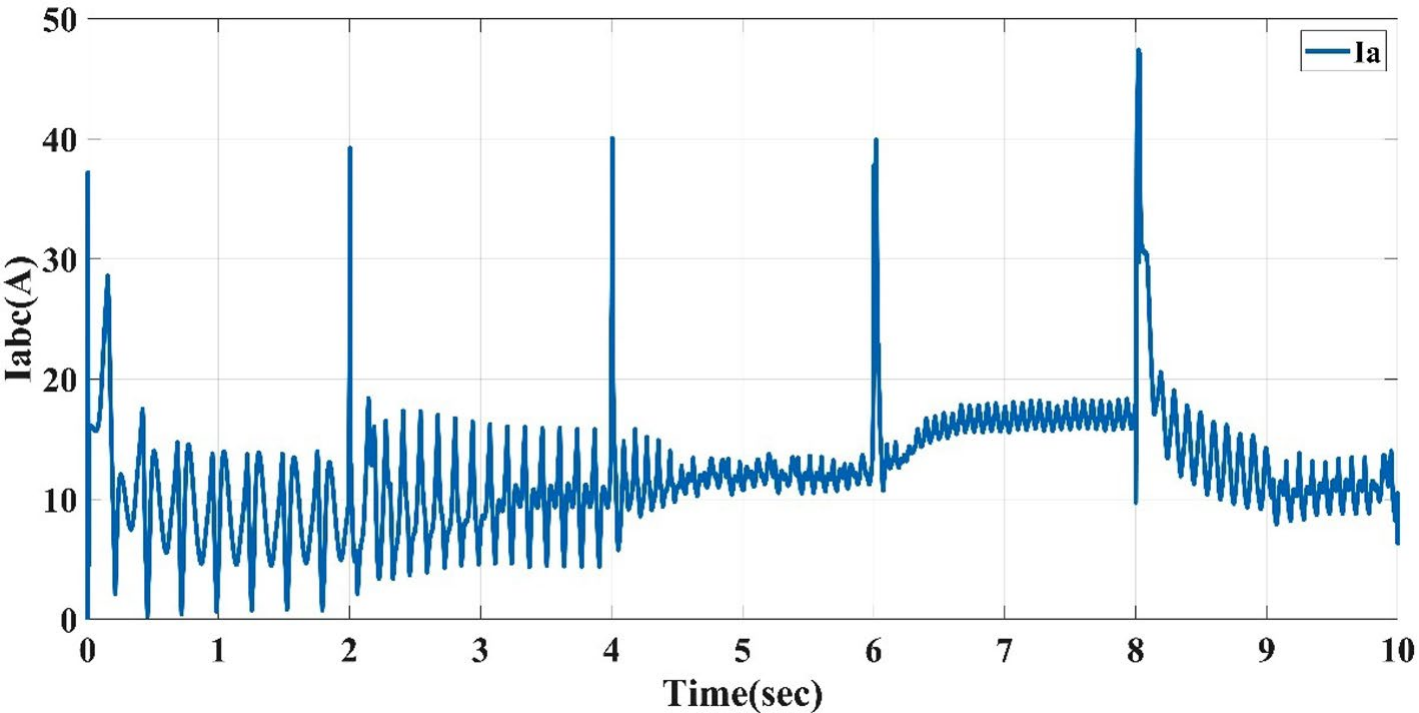
The AFPMSM RMS current at full load torque of 11 N m.

Also, this control system is the direct torque control type, which shows the torque response to the reference torque, as shown in Fig. 8 for speeds 75, 150, 225, 300, and 200 m/s each for 2 sec. It also shows the flux response to the reference flux as in Fig. 9, while Figs. 10 and 11 show the current drawn by the motor at full torque as instantaneous and RMS values, respectively. In the following subsection, the AFPMSM with DTC will be subject to testing for driving vehicles using two test systems.

**Figure 12:**
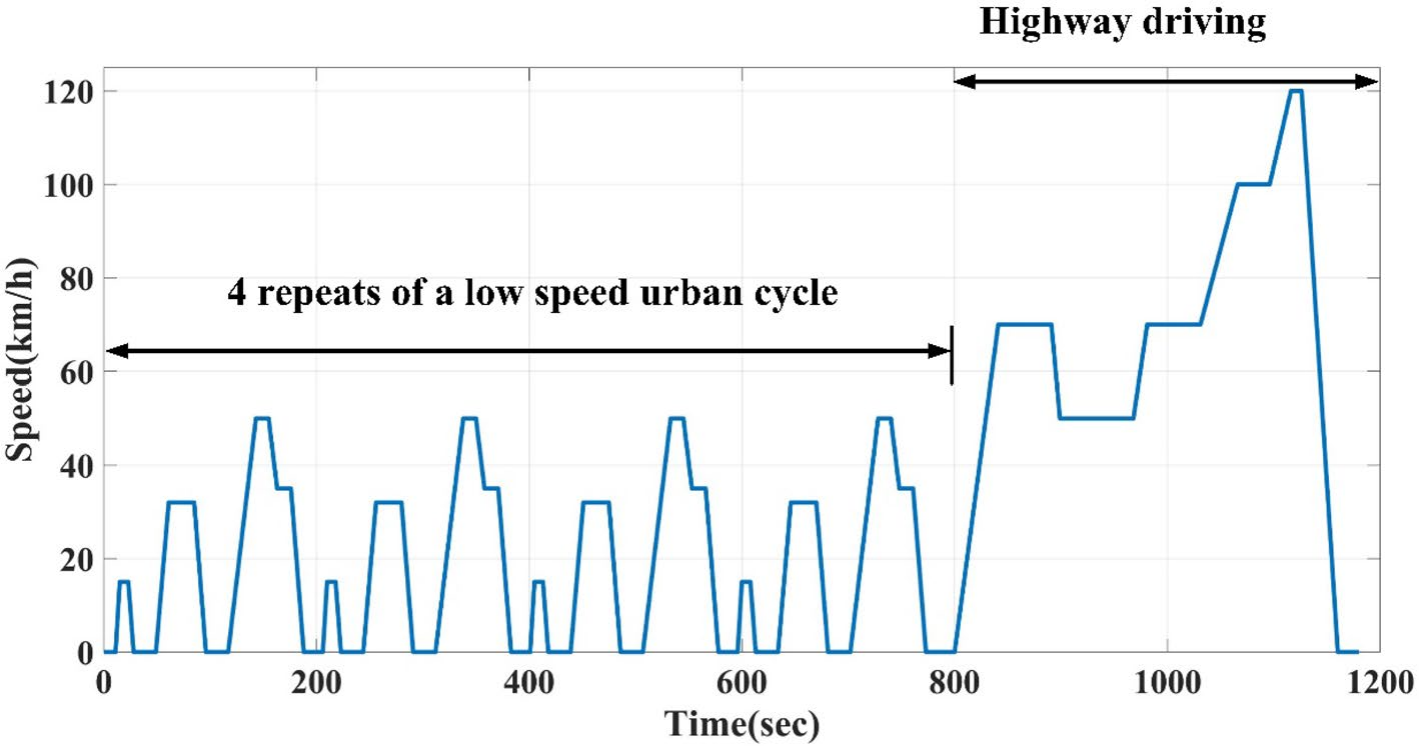
An example of NEDC driving cycle.

Light-duty vehicle type approval is based on the New European Driving Cycle (NEDC), which includes four repeats of a low-speed urban cycle and one highway drive with a 11,017-m distance, an 1180-s duration, and an average speed of 33.6 km/h. An example of NEDC Driving Cycle is shown in Fig. 12^27,28^.

Assume the EV tire diameter is 16 inch equal $16\times 2.54=40.64\mathrm{ cm}$, then the radius is $40.64/2=20.32\mathrm{ cm}$, the $\frac{\mathrm{km}}{\mathrm{h}}=\frac{1000}{2\pi \times 60\times {R}_{tire}}=\frac{1000}{2\pi \times 60\times 20.32\times {10}^{-2}}=13.054\mathrm{ rpm}.$

$${Gear}_{ratio}=\frac{motor \,rated \,speed }{max. \,speed (\frac{\mathrm{km}}{\mathrm{h}})\times 13.054\mathrm{ rpm}}$$

For New European Driving Cycle (NEDC) ${Gear}_{ratio}=\frac{300}{120\times 13.054 }=1/5.2216$.

For Environmental Protection Agency Highway Fuel Economy Test (HWFET) ${Gear}_{ratio}=\frac{ 300}{220\times 13.054 }=1/9.573$

**Figure 13:**
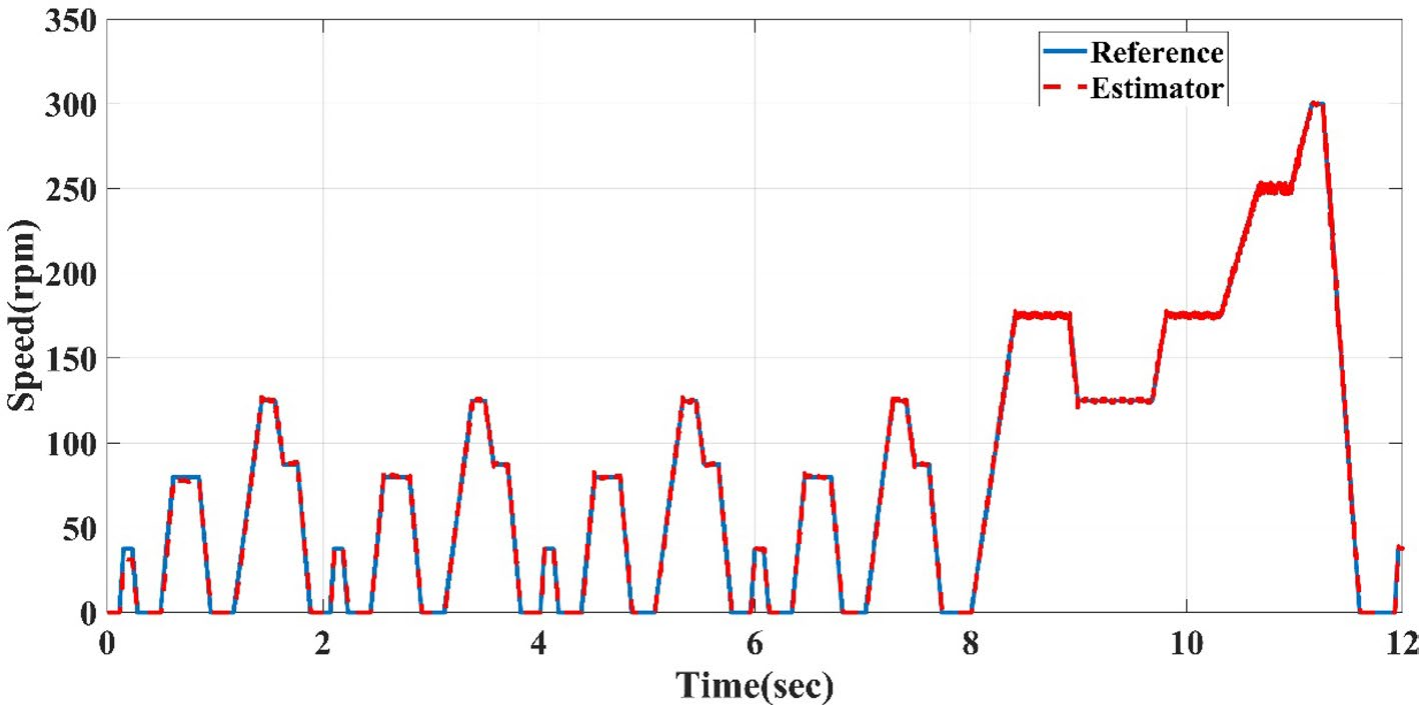
Reference NEDC speed cycle and straight-line guided by reference speed sensorless estimator of AFPMSM.

The straight-line guided by the reference speed sensorless estimator is tested to follow the reference NEDC speed cycle but with a time equal to 12 s instead of the standard time of the cycle, which is 1200 s. 12 s means the system response is faster than the actual NEDC by 100 times with high-performance and minimum transient and steady-state error, as shown in Fig. 13.

**Figure 14:**
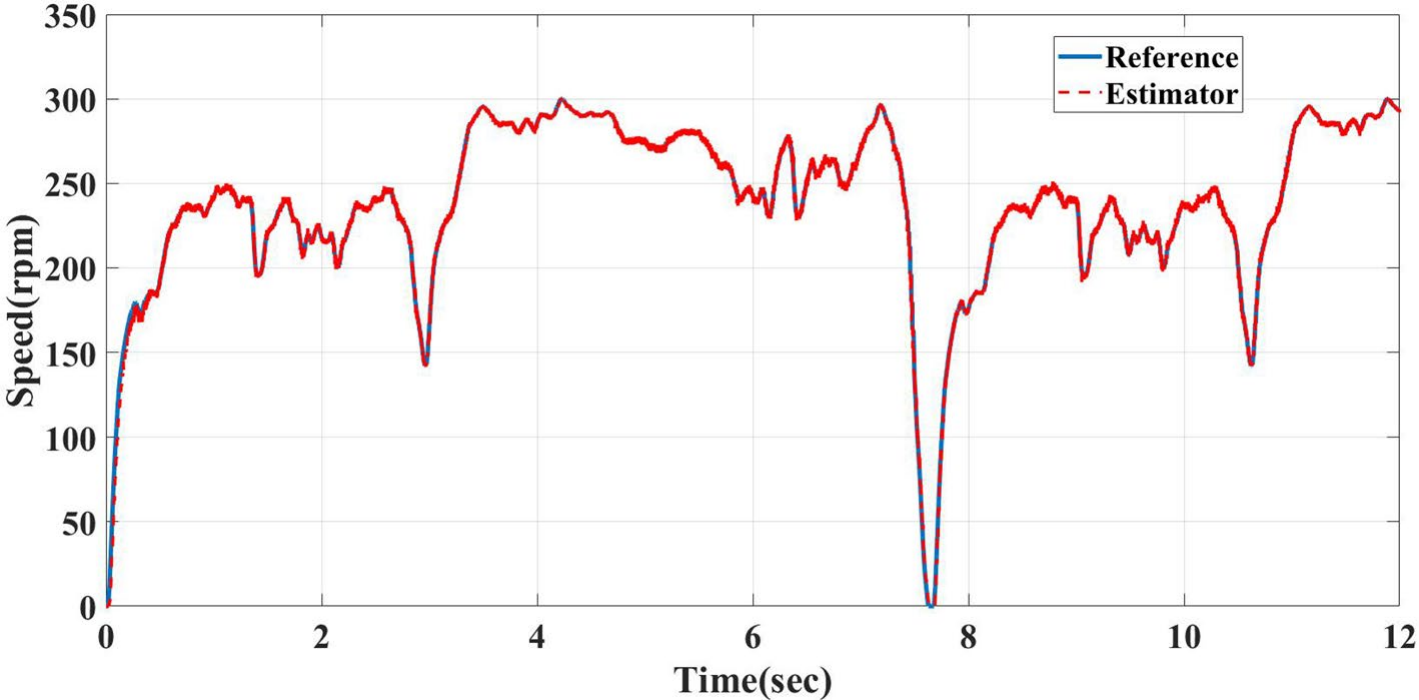
Reference HWFET speed cycle and straight-line guided by reference speed sensorless estimator of AFPMSM.

Environmental Protection Agency Highway Fuel Economy Test (HWFET) is modeled to drive cars by 16,503-m distance, 765-s duration, and 77.7 km/h average speed^28^. The straight-line guided by the reference speed sensorless estimator is tested to follow the reference HWFET speed cycle but with a time equal to 12 s instead of the standard time of the cycle, which is 1200 s. 12 s means the system response is faster than the actual NEDC by 100 times with high-performance and minimum transient and steady-state error, as shown in Fig. 14.

## Conclusion

The AFPMSM is suitable to use inside the vehicle tire, especially with a sensorless speed estimator. This paper presents the application of straight-line guided by reference speed sensorless estimator-based PI-DTC using SVPWM to control the axial flux motor speed for driving EVs. DTC ensures that the driver's safety and comfort are not jeopardized during driving. With a lower switching loss, lower THD rate, and better power factor (PF), SVPWM is the preferred solution. A sensorless estimator is used to reduce sensor inaccuracy while monitoring speed, and by using the sensorless estimator, the motor can also be installed inside the tire. Two driving cycles are employed to test the control system, and the results show that it performs admirably. NEDC and HWFET driving cycles are used to test the system control by time less than 100 times the actual time to test the coherence of the sensorless estimator. The controller results with the sensorless estimator for the NEDC and HWFET driving cycles present high-performance and minimum transient and steady-state error. The control system response to the proposed method reveals that the minimum overshoot is (0.11%), slight steady-state errors are (0.22%), and little rise time is (0.05 s) as a steady-state and transient performance.
